# ATP13A2 modifies mitochondrial localization of overexpressed TOM20 to autolysosomal pathway

**DOI:** 10.1371/journal.pone.0276823

**Published:** 2022-11-29

**Authors:** Yuta Hatori, Yukina Kanda, Saori Nonaka, Hiroshi Nakanishi, Takeo Kitazawa

**Affiliations:** 1 Department of Pharmaceutics, Faculty of Pharmacy, Yasuda Women’s University, Hiroshima, Japan; 2 Faculty of Pharmacy, Yasuda Women’s University, Hiroshima, Japan; 3 Department of Pharmacology, Faculty of Pharmacy, Yasuda Women’s University, Hiroshima, Japan; University of Nebraska-Lincoln, UNITED STATES

## Abstract

Mutations in ATP13A2 cause Kufor-Rakeb Syndrome (KRS), a juvenile form of Parkinson’s Disease (PD). The gene product belongs to a diverse family of ion pumps and mediates polyamine influx from lysosomal lumen. While the biochemical and structural studies highlight its unique mechanics, how PD pathology is linked to ATP13A2 function remains unclear. Here we report that localization of overexpressed TOM20, a mitochondrial outer-membrane protein, is significantly altered upon ATP13A2 expression to partially merge with lysosome. Using Halo-fused version of ATP13A2, ATP13A2 was identified in lysosome and autophagosome. Upon ATP13A2 co-expression, overexpressed TOM20 was found not only in mitochondria but also within ATP13A2-containing autolysosome. This modification of TOM20 localization was inhibited by adding 1-methyl-4-phenylpyridinium (MPP^+^) and not accompanied with mitophagy induction. We suggest that ATP13A2 may participate in the control of overexpressed proteins targeted to mitochondrial outer-membrane.

## Introduction

ATP13A2 belongs to the diverse family of molecular pumps (P-type ATPases) [1, 2] and recessive mutations have been identified in patients affected by Kufor-Rakeb Syndrome (KRS), a juvenile form of familial Parkinson’s Disease (PD) [3], which is characterized by cognitive impairment, myoclonus [4], and dementia [5]. Loss of ATP13A2 functions have been further associated with broad spectrum of neurodegenerative states, i.e., early-onset neuronal ceroid lipofuscinosis [6], hereditary spastic paraplegia [7], and amyotrophic lateral sclerosis-like phenotype [8].

In the aim of elucidating PD etiology, a series of genes responsible for the monogenic forms (PARK genes) have been characterized, recognizing mitochondrial and proteostatic dysfunctions as a main pathogenetic mechanism underlying PD development [9, 10]. Among 23 PARK genes, ATP13A2 (PARK9) is rather atypical in that the gene product is primarily present in lysosome while others have direct contacts with mitochondria. The molecular function of human ATP13A2 had been critically elusive until recent biochemical and structural studies illuminated its ability to transport polyamines from the lumenal to cytoplasmic side across the biological membrane [11–13]. The published structures unveil distinct intermediate states of ATP13A2 including complexes with polyamines bound in the electronegative cavity passing through the transmembrane α-helical bundle [14, 15]. Polyamines were shown to stimulate the ATPase cycle of ATP13A2 and indeed translocated across the biological membrane in ATP- and ATP13A2-dependent manner [11]. These key evidences have founded the biochemical nature of ATP13A2 as a polyamine pump.

While the shape of ATP13A2 is emerging at atomic resolution, understanding of its role in cell physiology is limited. The most fundamental and ultimate question is “how is the deficiency of ATP13A2 linked to PD development?” Reflecting pathologic features of KRS, ATP13A2 knockout mouse was characterized by rupture in dopaminergic neurons, sensorimotor impairment, and Lewy body accumulation [16, 17]. Development of ceroid lipofuscinosis with accumulated mitochondrial protein was also reported [18]. At cellular level, a number of studies have implicated ATP13A2 in lysosomal function. ATP13A2-deficient fibroblasts from KRS patients exhibited deficits in lysosomal acidification and autophagic processes [19]. Like other PARK genes, ATP13A2 inactivation results in mitochondrial dysfunction [20, 21] which is at least in part attributed to a defect in autophagy [21]. In human neuroblastoma SH-SY5Y cells with ATP13A2 deficiency, the level of lysosomal cathepsin D was downregulated, reflecting reduced proteolytic activity in lysosome [22]. Consistently, other studies pointed to the impacts of ATP13A2 on the cellular proteostasis via mTORC1-TFEB pathway [23], endolysosomal cargo sorting [24], clearance of mitochondria-derived dsDNA [25], autophagic regulation of iron homeostasis [26], and lysosomal HDAC6 [27]. These studies collectively shed a light on an important role of ATP13A2 in autophagic/lysosomal processes. For thorough understanding of the underlying mechanism, further investigation into the cellular impact of ATP13A2 activation/inactivation is required.

Here we report localization analyses of ATP13A2 utilizing various fluorescent organelle-specific proteins and Halo tag fusion which allowed flexible strategy of live-cell imaging. When proteins at the mitochondrial outer membrane(mCerry-TOM20-N,GFP-Mff) were overexpressed, their localizations changed to merge with ATP13A2-containing autolysosome. Importantly, this alteration was dependent on ATP13A2 overexpression, pointing to an important role of ATP13A2 in maintaining normal proteostasis or membrane targeting at mitochondrial outer membrane. We further found that MPP^+^, a PD-inducing neurotoxin, interfered with ATP13A2-dependent modification of mCherry-TOM20-N localization. Coexpression of ATP13A2 and Tom20 did not alter the frequency of mitophagic cells, indicating that autolysosomal localization of overexpressed Tom20 was not attributed to increased mitophagic activity. These findings provide novel information regarding the cellular function of ATP13A2 and potentially contributes to further understanding of PD etiology.

## Materials and methods

### Antibodies

The antibodies were obtained from the following manufacturers; polyclonal anti-ATP13A2 antibody produced in rabbit (Sigma-Aldrich, A9607), polyclonal anti-ATP13A3 antibody produced in rabbit (Sigma-Aldrich, SAB1302984), monoclonal anti-β-actin antibody produced in mouse (Sigma-Aldrich, A5441), monoclonal anti-Tom20 antibody produced in mouse (F-10, Santa Cruz, sc-17764), polyclonal anti-rabbit IgG (H+L) Alexa Fluor 568 conjugate (Thermo Fisher Scientific, A11011) and polyclonal anti-rabbit IgG (H+L) Alexa Fluor 488 conjugate (Thermo Fisher Scientific, A11008).

### Plasmids

The plasmids are gifts from the following researchers; Su9-mCherry-GFP [28] from Dr. Miho Iijima, mCherry-TOMM20-N-10 from Dr. Michael Davidson (Addgene plasmid #55146), GFP-Mff from Dr. Gia Voeltz (Addgene plasmid #49153 [29]), pLPCX mito Grx1-roGFP2 from Dr. Tobias Dick (Addgene plasmid # 64977 [30]), Lamp1-RFP from Dr. Walther Mothes (Addgene plasmid # 1817 [31]), pEGFP-LC3 (human) from Dr. Toren Finkel (Addgene plasmid #24920 [32]). Constructions of Mito Grx1-roGFP2, PMP2-Grx1-roGFP2, LifeAct7-Grx1-roGFP2, Keratin-Grx1-roGFP2 and Sec61β-Grx1-roGFP2 were previously described [33, 34].

cDNA of human ATP13A2 (variant 1, NCBI accession number NM_022089.4) was *de novo* synthesized via GenScript and subcloned into pcDNA3.1+C-eGFP, resulting in ATP13A2-GFP, respectively. ATP13A2 was then amplified by PCR and inserted into pHTC HaloTag CMV-neo vector, generating ATP13A2-Halo construct. In this plasmid, the fusion protein is attached with C-terminal protease-recognition site (TEV) and Halo tag.

For constructing native form of human Tom20, 436ggc438 in the coding region of mCherry-TOMM20-N-10 was replaced with stop codon (tga) using QuikChange II Site-Directed Mutagenesis Kits (Agilent Technology) and a set of complementary oligonucleotides; Tom20F_g436t_c438a, 5’-gatccccgctacctcattccacatcatcttcagccaagc-3’; Tom20R_g436t_c438a, 5’-gcttggctgaagatgatgtggaatgaggtagcggggatc-3’. DNA sequences of generated constructs were validated by Sanger sequencing.

For transfection into mammalian cells, DNA plasmids were propagated in *Escherichia coli* DH5α (TAKARA) or XL10-Gold (Agilent Technology) strain and purified using Plasmid Plus Midi Kit (QIAGEN).

### Cell culture

HeLa cells were cultured in Dulbecco’s Modified Eagle Medium (DMEM) with 10% FBS, 50U/ml penicillin and 50 μg/ml streptomycin at 37°C and 5% CO_2_. DMEM, FBS and streptomycin were purchased from Thermo Fisher Scientific. Plasmid transfection was performed using Lipofectamine 3000 and Opti-MEM (Thermo Fisher Scientific). For some experiments, 100 μM or 500 μM 1-methyl-4-phenylpyridinum (MPP^+^) was added to culture medium 24 h after plasmid transfection. Stably transfected cells were obtained by treating cells with 500 μg/mL geneticin (Thermo Fisher Scientific) over 10 days.

### Immunocytochemistry

Cells were fixed with 4% paraformaldehyde, permeabilized with 0.2% Triton X-100 in PBS and blocked by 3% BSA. Cells were then sequentially treated with primary antibodies (4°C, overnight) and secondary antibodies (room temperature, 3h). Cells were then mounted with Fluoromount-G containing DAPI (SouthernBiotech). Cells were visualized by confocal microscopy using FV1000 (Olympus, Japan) equipped with a temperature- and CO_2_-controlled stage chamber. Images were processed using Image J [35] with Fiji package [36].

### Detection of Halo-fused protein separated on SDS gels

HEK cells were transfected with pHTC/ATP13A2-Halo. Cells stably expressing ATP13A2-Halo were selected by using 500 μg/ml G418. Selected cells were mechanically collected without trypsin treatment, washed with PBS, and resuspended in hypotonic buffer (10 mM HEPES, pH7.0) with Protease Inhibitor Cocktail (Nakalai). After lysing cells by homogenization, the composition of the cell suspension was adjusted to 25 mM HEPES, pH7.0, 150 mM NaCl and 10% glycerol. Debris as well as nuclei were removed by centrifugation at 5,000×g for 20 min. Supernatant was further fractionated by ultracentrifugation at 40,000×g for 1 hr. Pelleted microsome was resuspended in PBS. Protein concentration was determined by bicinchoninic acid assay. Twenty five μg of microsomal protein was incubated with 10 μM TMR Halo-ligand or 2 μM Oregon Green Halo-ligand (Promega) for 20 min at room temperature. Before electrophoretic separation of proteins, each sample was combined with one third volume of 4×Laemmlli sample buffer containing 50 mM dithiothreitol. Proteins were then resolved by SDS-PAGE and fluorescence on gels were imaged by LAS3000 luminoimage analyzer (Fujifilm).

### Co-immunoprecipitation assay

To explore potential interaction between overexpressed ATP13A2-Halo and mCherry-Tom20, pull-down assay was performed. Hela cells stably transfected with ATP13A2-Halo were plated on 10 cm dish and additionally transfected with mCherry-TOM20-N-10. Two days after transfection, cells were washed with ice-cold PBS and lysed in 20 mM HEPES buffer pH7.5 containing 0.15 M NaCl, 0.5% (w/v) Triton-X 100, and protease inhibitor cocktail (Nacalai Tesque). Collected lysate was cleared by centrifugation at 13,000×g for 30 min. Primary antibodies (either anti-ATP13A2 or normal IgG) were added, followed by overnight incubation at 4°C. Antibody-antigen complex was adsorbed to Dynabeads Protein G (Thermo Fisher Scientific) and magnetically sedimented. The beads were washed with lysis buffer (the concentration of TritonX-100 was adjusted to 0.1% w/v) five times. After completely removing supernatant, SDS sample buffer was added to protein G beads to resolve adsorbed immune complex. Supernatant was recovered, supplemented with 10 mM dithiothreitol, heat-denatured at 100°C for 5 min, and subjected to western blotting.

Proteins were separated on SDS-polyacrylamide gels containing either 5.5% (ATP13A2 detection) or 10% (Tom20 detection) acrylamide, transferred to PVDF membranes, blocked using blocking one reagent (Nacalai Tesque), and incubated with primary antibodies at 4°C overnight. The membranes were then reacted with secondary antibodies at room temperature for 1 hour. Chemiluminescence was detected using LAS-4000 luminoimageanalyzer (Fujifilm).

## Results and discussion

**Fig 1:**
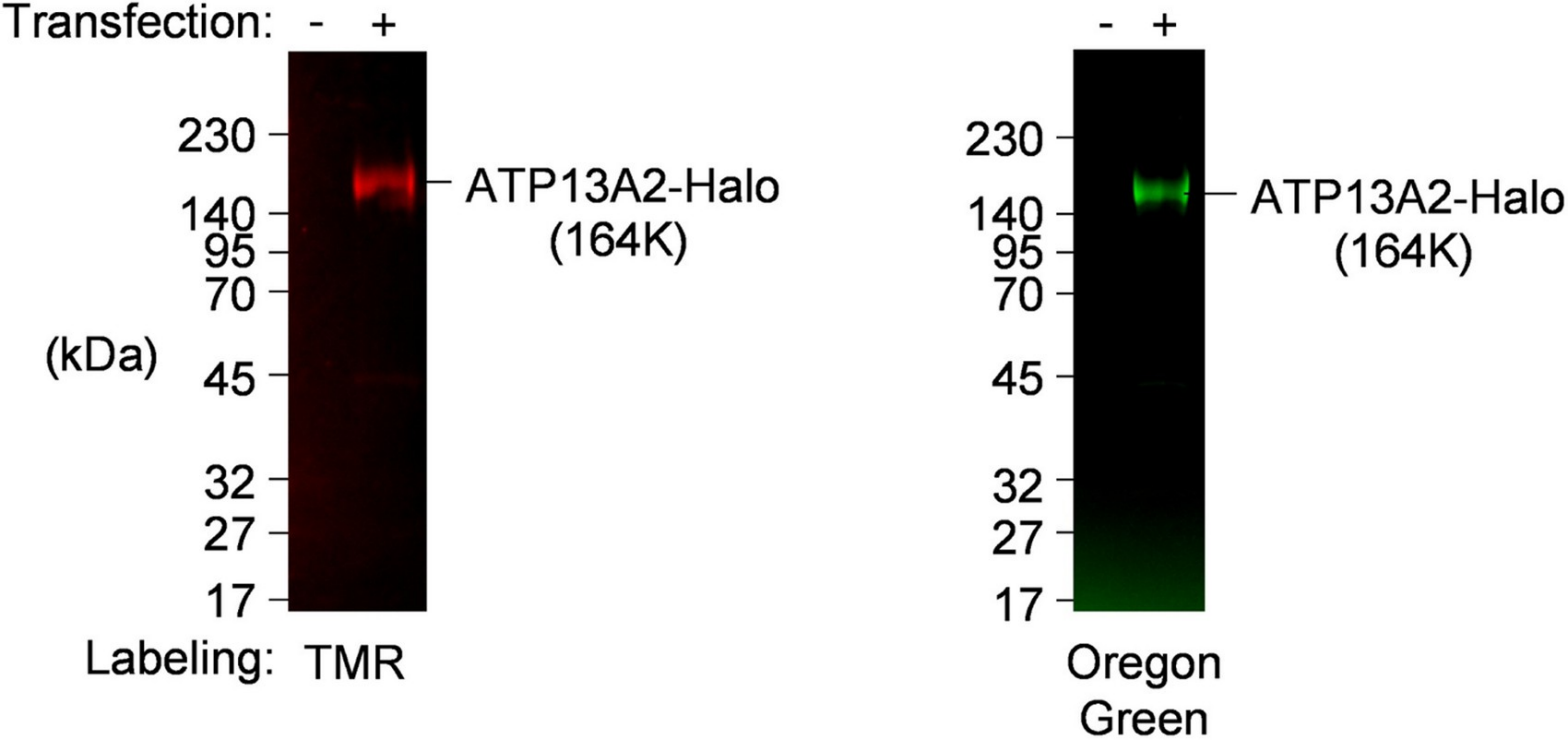
Construction and expression of ATP13A2-Halo. Halo tag was C-terminally fused with human ATP13A2. Pilot expression in HEK cells produced expected size of ATP13A2-Halo protein (164K). Stable cells were harvested and microsomes were prepared. ATP13A2-Halo was specifically labeled *ex vivo* with TMR-ligand (left) or Oregon green (right), followed by SDS-PAGE and fluorescence imaging.

**Fig 2:**
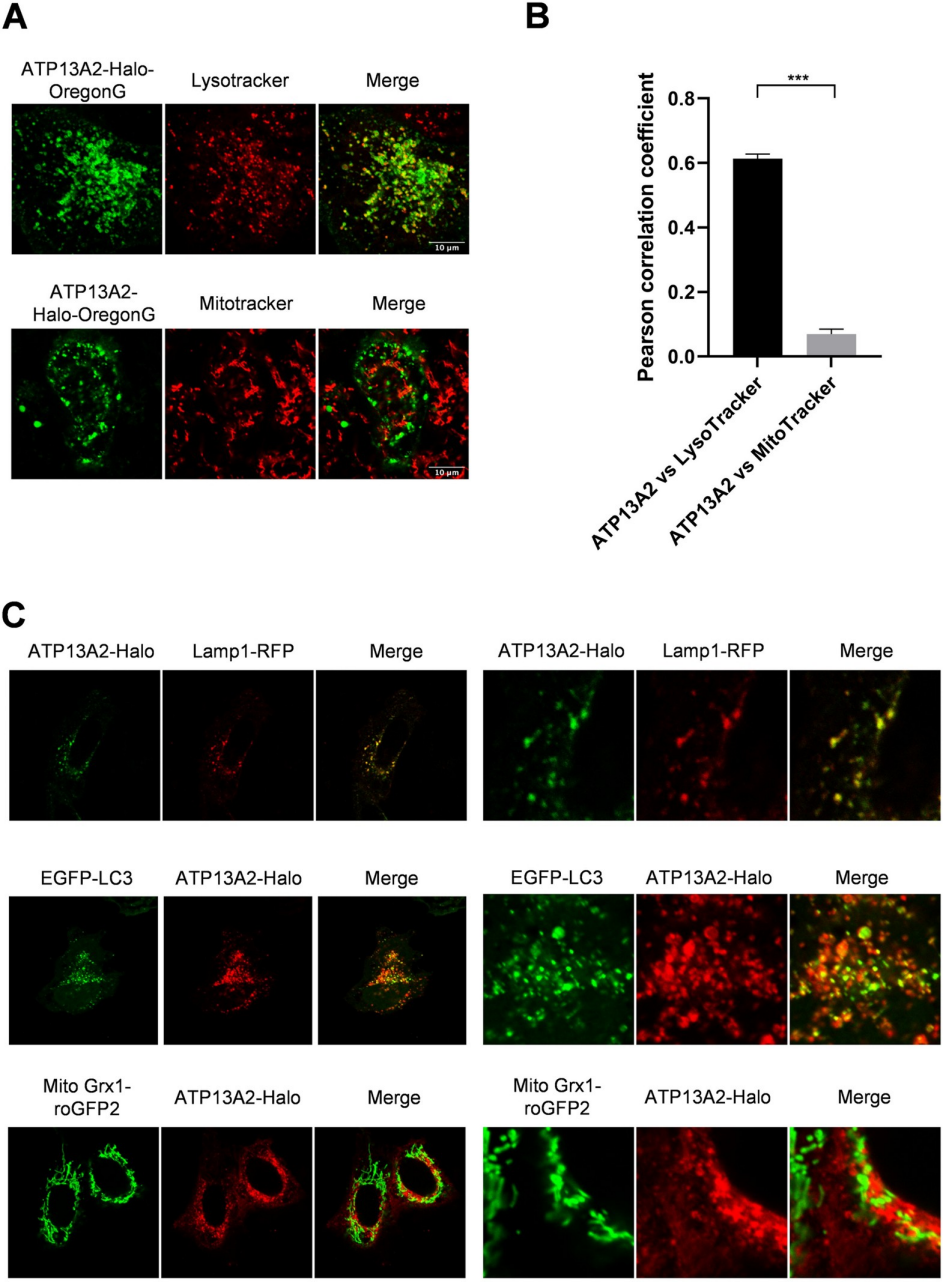
Intracellular localization of ATP13A2-Halo in HeLa cells. **(A)** Images of live cells stained with indicated fluorophores. ATP13A2-Halo was labeled with Oregon Green by utilizing Halo link technology. Scatter plots are also shown in S2 Fig **(B)** Pearson correlation coefficients of ATP13A2-Halo and Lysotracker or Mitotracker. Data represent averages of n = 10 samples with S.E.M. (bars). Statistical analysis was performed using Student’s t test: *p < 0.05, ** *p* < 0.01, *** *p* < 0.001. **(C)** Organelle-specific proteins were cotransfected with ATP13A2-Halo. For obtaining high resolution images, cells were fixed and subjected to immunostaining using anti-ATP13A2 antibody.

In order to analyze the intracellular localization of ATP13A2 at fine spatiotemporal resolution, we constructed recombinant ATP13A2 fused with C-terminal Halo tag (ATP13A2-Halo) which facilitates protein detection and purification. Halo-directed labeling enabled specific detection of ATP13A2-Halo. Overexpressed protein exhibited a theoretical mass of full length protein (164KDa) and no degraded or immatured protein was observed (Fig 1). Using this construct, first, we sought to confirm distribution pattern of ATP13A2-Halo in HeLa cells. ATP13A2 was previously reported to reside in lysosome and autophagosome. Consistently, Oregon Green-labeled ATP13A2-Halo was identified as vesicular structures seemingly overlapping with or surrounding Lysotracker-stained acidic compartments (Fig 2A, top). Meanwhile, no contact with mitochondria was observed (Fig 2A, bottom). Pearson correlation coefficients (PCCs) were 0.61±0.015 and 0.07±0.015, respectively (Fig 2B and S1 Fig). The observed pattern matches lysosomal localization, which was further tested using a series of fluorescent markers listed in Table 1. ATP13A2-Halo exhibited complete overlap with lysosome marker and partially colocalized with autophagosome marker, indicating the presence at lysosome/autolysosome membranes (Fig 2C), as previously reported for GFP-fused ATP13A2 [3]. Thereby lysosomal/autolysosomal localization of ATP13A2 was preserved without apparent effect of Halo-fusion.

**Table 1:** List of organelle markers used in the study and summary of the results.

Construct name	targeted organelle	Influence of ATP13A2 on distribution pattern	colocalization with ATP13A2-Halo	Reference
Mito Grx1-roGFP2	Mitochondria, IMS/inner membrane	No	No	30
mCherry-Tom20-N	Mitochondria, outer membrane	Yes (from tubular to particulate)	Partial	#55146*
GFP-Mff	Mitochondria, outer membrane	Yes (from tubular to particulate)	Partial	29
Lamp1-RFP	Lysosome membrane	No	Complete	31
EGFP-LC3	Autophagosome membrane	No	Partial	32
Sec61b-Grx1-roGFP2	ER membrane	No	No	33

*Addgene number

**Fig 3:**
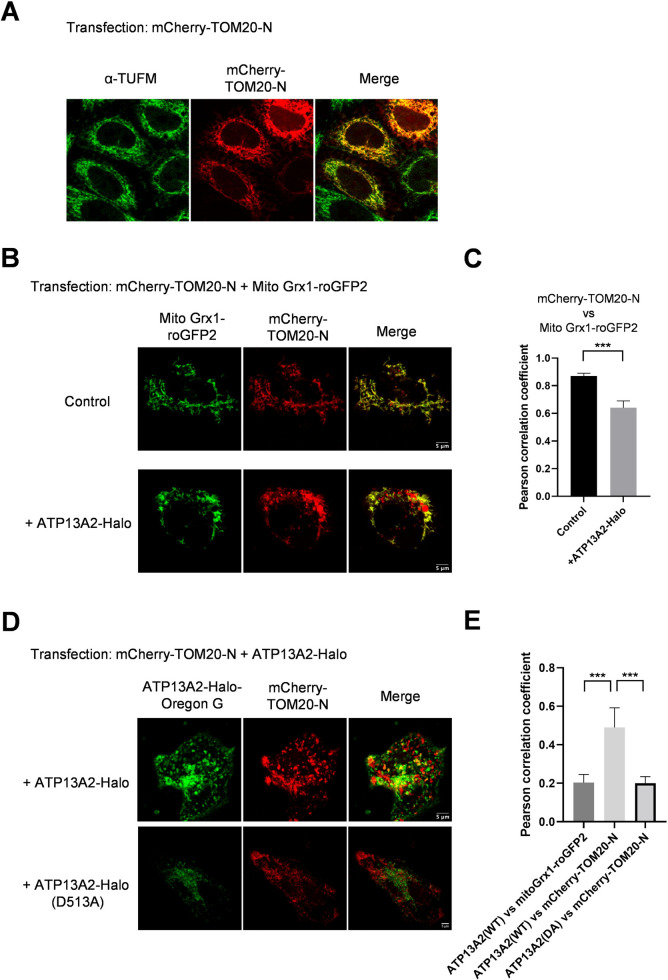
ATP13A2 overexpression altered the intracellular distribution of mCherry-TOM20-N (mitochondrial outermembrane, MOM). **(A)** MOM protein, mCherry-TOM20-N, was overexpressed in HeLa cells and mitochondrial localization was confirmed by immunostaining; TOM20 exhibited complete colocalization with the endogenous mitochondrial protein, TUFM. **(B)** Mitochondria was visualized by mitochondrial intermembrane space (IMS)/matrix marker, mito Grx1-roGFP2. Upon co-overexpression of ATP13A2, substantial portion of mCherry-TOM20-N appeared as particulate structures. Scatter plots are also shown in S3 Fig **(C)** Pearson correlation coefficients of mCherry-TOM20-N and mito Grx1-roGFP2 are shown. Data represent averages of n = 10 samples with S.E.M. (bars). Statistical analysis was performed using Student’s t test: *p < 0.05, ** *p* < 0.01, *** *p* < 0.001. **(D)** mCherry-TOM20-N and ATP13A2-Halo were coexpressed and their localizations were analyzed by live-cell imaging. TOM20 showed mixed localization pattern with mitochondria-like tubular shapes and particulate structures. The latter signals substantially merge with ATP13A2-positive vesicles. Catalytically dead mutant of ATP13A2 (D513A) produced no effect on the localization pattern of mCherry-TOM20-N. Scatter plots are also shown in S4 Fig **(E)** Pearson correlation coefficients of indicated proteins are shown. Data represent averages of n = 10 samples with S.E.M. (bars). Statistical analysis was performed using Student’s t test: *p < 0.05, ** *p* < 0.01, *** *p* < 0.001.

Majority of PD-associated genes found so far have been linked to management of mitochondrial quality, which drove us to further test colocalization of ATP13A2 with various mitochondrial proteins. In addition to matrix/inner membrane-directed marker (Fig 2; mito Grx1-roGFP2), we used another fluorescent marker, mCherry-TOM20-N, targeted to mitochondrial outer membrane (MOM). When singly transfected, mCherry-TOM20-N exhibited tubular distribution pattern and precisely overlapped with endogenous mitochondrial protein, TUFM (Fig 3A), as well as exogenously transfected marker of mitochondrial matrix (mito Grx1-roGFP2, Fig 3B, top), ensuring mitochondrial localization. Unexpectedly, upon co-overexpression of ATP13A2-Halo, mCherry-TOM20-N changed their localization pattern from tubular to partially particulate distribution (Fig 3B, bottom). Accordingly, PCC between mCherry-TOM20-N and the mitochondrial matrix marker (mito Grx1-roGFP2) significantly dropped from 0.87±0.021 to 0.64±0.050, pointing to partial discolocalization (Figs 3C and S3). Of particular interest, these particulate populations of mCherry-TOM20-N colocalized with ATP13A2-positive vesicles, i.e., lysosome/autolysosome (Fig 3D, top). This resulted in higher PCC of ATP13A2-Halo vs mCherry-TOM20-N (0.49±0.102) compared to the value vs mitochondrial matrix marker (mito Grx1-roGFP2; 0.20±0.043, Figs 3E and S4). Catalytically dead mutant (D513A) failed to produce apparent effect on the localization of mCherry-TOM20-N, suggesting that the observed effect depends on catalytic activity of ATP13A2 (Fig 3D, bottom and Fig 3E).

To determine whether ATP13A2 action on a localization pattern is specific to TOM20 or common for other MOM proteins, localization of EGFP-Mff was also analyzed. As observed for mCherry-TOM20-N, EGFP-Mff was present within ATP13A2-containing vesicles (S5 Fig). Within 9 organelle-specific proteins tested in the study, only mCherry-TOM20-N and GFP-Mff were influenced by ATP13A2, suggesting that this ATP13A2 effect may be specific to MOM proteins. These observations are not inconsistent to the idea that ATP13A2 may be involved in regulating localizations of MOM proteins.

**Fig 4:**
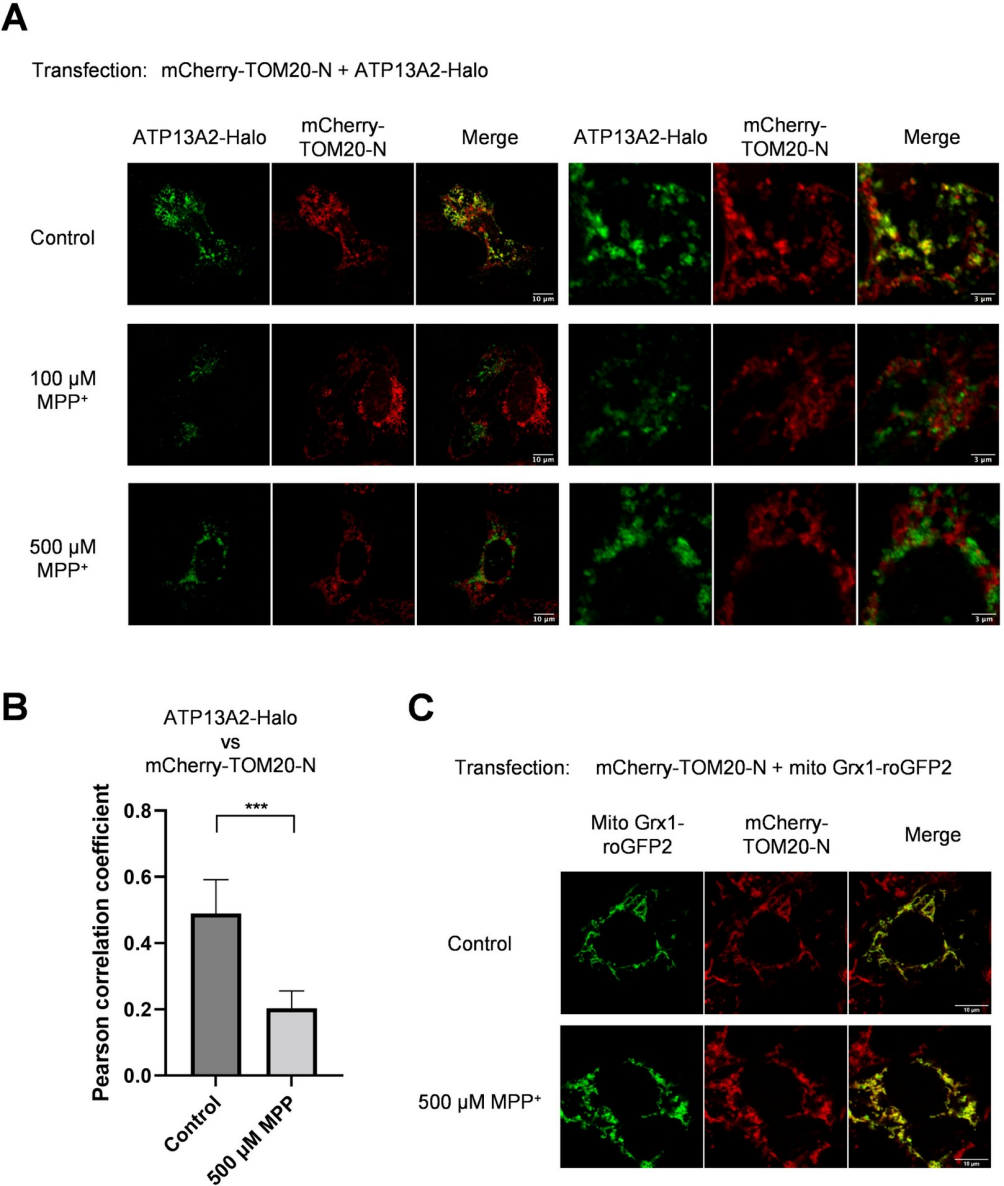
ATP13A2-dependent sorting of mCherry-Tom20-N is sensitive to MPP^+^ treatment. **(A)** HeLa cells were cotransfected with mCherry-TOM20-N and ATP13A2-Halo. One day after transfection, MPP^+^ was added to the culture medium and cells were further incubated for 24 hours, followed by immunostaining. After MPP^+^ treatment, no colocalization was observed for ATP13A2 and TOM20. Scatter plots are also shown in S6 Fig **(B)** Pearson correlation coefficients of ATP13A2-Halo and mCherry-TOM20-N are shown. Data represent averages of n = 10 samples with S.E.M. (bars). Statistical analysis was performed using Student’s t test: *p < 0.05, ** *p* < 0.01, *** *p* < 0.001. **(C)** Together with mCherry-TOM20-N, mito Grx1-roGFP2 was transfected for assessing mitochondrial shapes in the presence of MPP^+^.

Additionally, we tested the effect of MPP^+^ which exerts neurotoxicity presumably through intervention in mitochondrial electron transport chain. More recently, impaired autophagic degradation by mild MPP^+^ exposure was reported [37]. In a condition with acute toxicity (500 μM MPP^+^, 24 hr), ATP13A2 failed to induce colocalization of mCherry-TOM20-N and ATP13A2-Halo (Fig 4A). PCC between ATP13A2-Halo and mCherry-TOM20-N dropped from 0.49±0102 to 0.20±0.052 upon MPP^+^ treatment (Figs 4B and S6).

Considering the possibility that overexpressed mCherry-TOM20-N was trapped on mitochondria, mitochondria morphology in MPP^+^-treated culture was visualized using mito Grx1-roGFP2. After MPP^+^-treatment, mCherry-TOM20-N was completely merged with mito Grx1-roGFP2 (Fig 4C). This suggests that mCherry-TOM20-N was present at mitochondria even with ATP13A2 expression and potentially insensitive to ATP13A2-dependent action.

Considering the possibility that co-overexpression of ATP13A2 and Tom20 may trigger mitochondrial damage and autophagic processing of impaired mitochondria (mitophagy), we sought to detect mitophagic cells using a mitophagy biosensor, matrix-targeted Su9-

**Fig 5:**
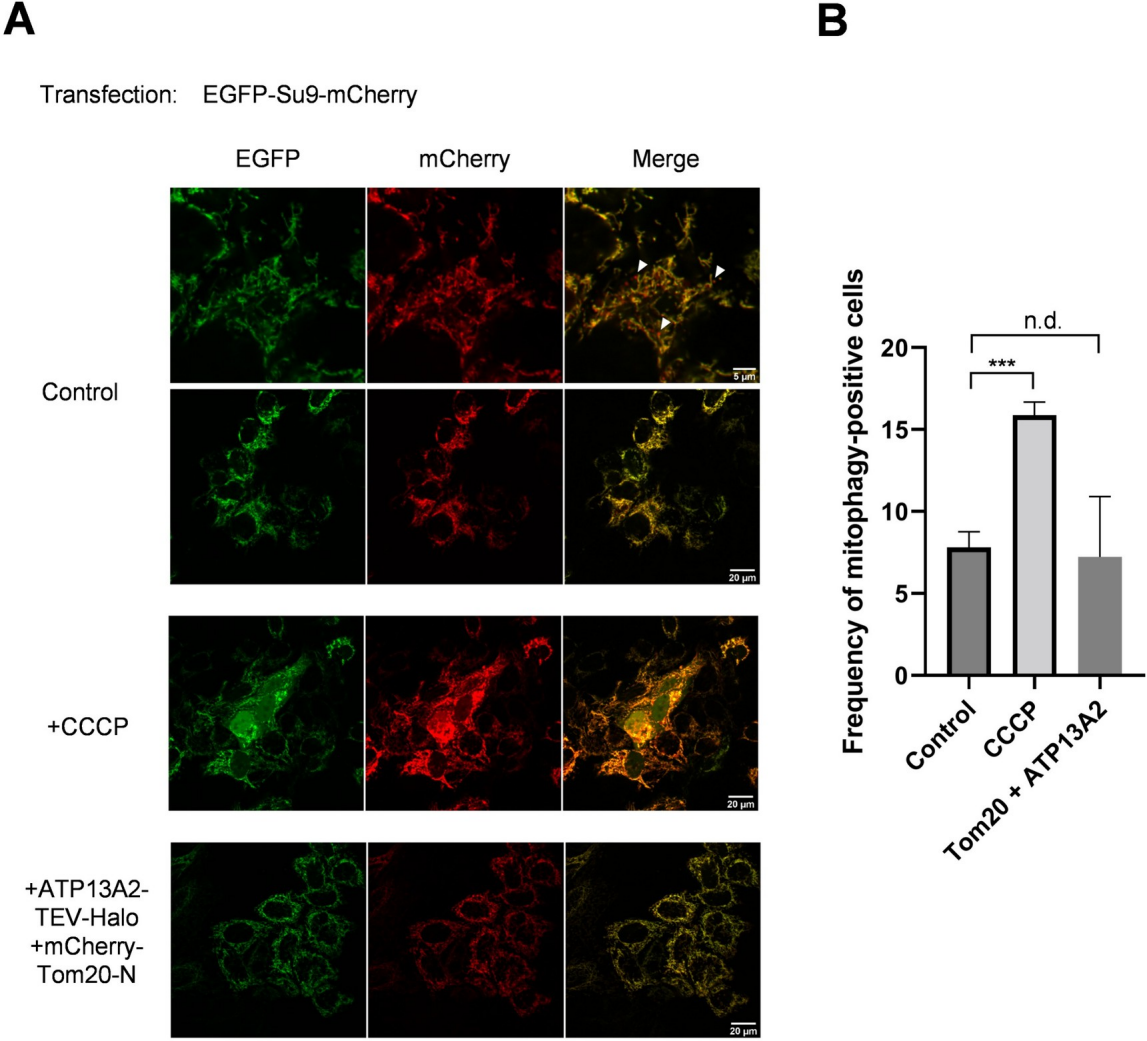
The number of mitophagic cells is not affected by co-expression of ATP13A2-Halo and Tom20. HeLa cells stably overexpressing ATP13A2-Halo were additionally transfected with plasmids carrying Tom20 and Su9-mCherry-GFP. Two days after transfection, the cells were analyzed by confocal microscopy. The mitophagic cells are defined as those exhibiting mCherry-positive and GFP-negative particles. White arrow heads indicates representative mitophagic particles. As a positive control condition, cells were treated with 50 μM 3-chlorophenylhydrazone (CCCP). (B) The numbers of mitophagic cells were counted and shown as frequencies (%). Values are average ± S.E.M. (n = 50 cells). Statistical analysis was performed using Student’s t test: *p < 0.05, ** *p* < 0.01, *** *p* < 0.001, “n.d.” difference is not significant.

mCherry-GFP [28, 38–40]. Mitochondrially targeted pool of Su9-mCherry-GFP emits fluorescence from both mCherry and GFP fluorophores. During mitophagy, mitochondria is transported to lysosomes and subjected to acidic pH, resulting in selective loss of GFP fluorescence. The results show that the number of mitophagic cells was not significantly altered by coexpression of ATP13A2 and Tom20 (Fig 5), again suggesting that mitochondrial damage is not prominent in this condition.

**Fig 6:**
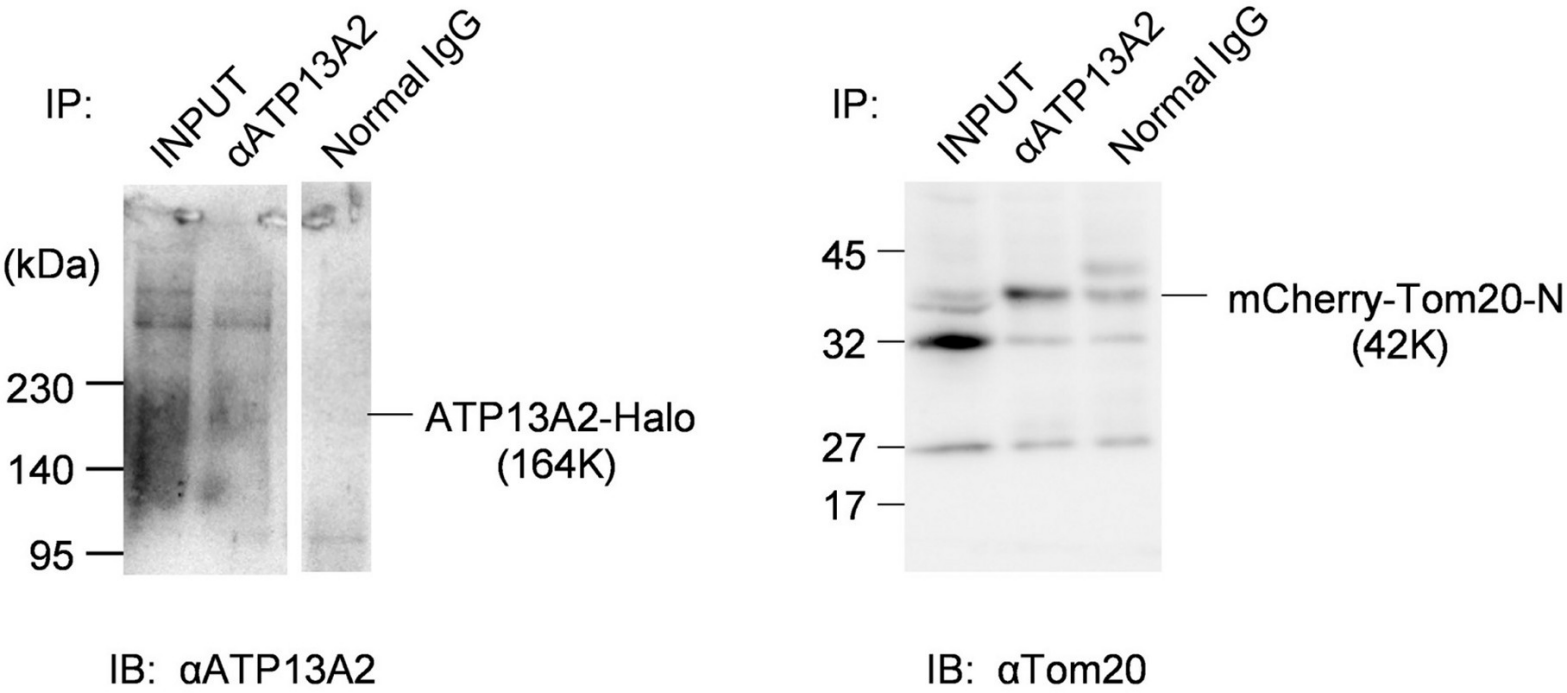
Co-IP detected interaction between ATP13A2-Halo and mCherry-Tom20-N. Cells co-expressing ATP13A2-Halo and mCherry-Tom20-N were assayed for protein interaction. Immunoprecipitation was performed using anti-ATP13A2 antibody and co-precipitated mCherry-Tom20-N was detected. The original entire images of the membranes are shown in S7 Fig.

We explored possible interaction between overexpressed ATP13A2-Halo and mCherry-Tom20 by co-IP. ATP13A2-Halo was pulled down using specific antibody. The level of co-immunoprecipitated mCherry-Tom20 was markedly higher than the condition with normal IgG (Fig 6), pointing to significant interaction between overexpressed ATP13A2 and mCherry-Tom20.

Overall, this study demonstrated for the first time that localization of overexpressed TOM20, a mitochondrial protein, is influenced by expressing a lysosomal pump, ATP13A2. ATP13A2 belongs to P5B subgroup of P-type ATPase family and is present within lysosomal/autolysosomal pathway. ATP13A2 expression altered localization of mCherry-TOM20-N to merge with autolysosome. This process, sensitive to MPP^+^, may potentially contribute to maintaining the local proteostasis on the outermembrane of mitochondria.

Although localization of ATP13A2 has been limited to lysosome/autolysosome, substantial evidences have linked ATP13A2 function to mitochondria physiology. Grünewald and her coworkers observed decreased level of mitochondrial ATPase synthesis in fibroblast derived from KRS-affected patients [20]. Defective energy metabolism in mitochondria was further attributed to deficit in autophagy [21]. As represented by these early works, many studies pointed to involvement of ATP13A2 in mitochondrial and/or autophagic processes. In this line, it may not be very surprising that ATP13A2 mediates sorting of mitochondria-targeted protein to autolysosome. However, how this could be achieved by this polyamine pump is still elusive. For answering this question, evidence linking polyamine homeostasis and autophagy is awaited. Polyamines was reported to increase the lifespan of worms and flies and improve neural functions via autophagy induction [41, 42] and, in human, blood level of polyamines serves as a potential PD biomarker [43]. More specifically, spermidine was shown to enhance respiratory capacity in mouse hippocampus and, in flies, this polyamine action requires functional autophagy as well as the mitophagy mediators Pink1 and Parkin, pointing to an important role of polyamine in mitochondrial proteostasis [44]. Alternatively, the recently published study of P5A ATPase (yeast Spf1) might give a direct hint; Spf1 (yeast homologue of human ATP13A1) mediates resorting of mitochondrial proteins from ER membrane to the right destination by ATP-driven extraction of substrate polypeptides [45]. This is of interest considering that localization of mitochondrially targeted protein, mCherry-TOM20-N, in this study was also affected by ATP13A2. We think at least two alternative possibilities for explaining our results; (i) lysosomal proteins, when excessively overexpressed, may end up with nonspecifically perturbed membrane trafficking within a cell; (ii) similar to ATP13A1, ATP13A2 may contribute to membrane sorting of specific protein(s). The former possibility seems unlikely considering that localizations of other marker proteins were not affected by ATP13A2 overexpression. In addition, catalytically dead mutant, D498A, were successfully overexpressed to the WT level but did not affect localization of mCherry-TOM20-N. We expect that a unique role of ATP13A2 in mitochondrial proteostasis might emerge from further investigation into the molecular basis of the observed effect of ATP13A2 overexpression.

The current study demonstrated the impact of ATP13A2 expression on the localization of Tom20, where overexpressed Tom20 was apparently sorted to autolysosomal pathway. This is particularly of interest considering the pertinent role of TOM complex in the PD etiology. TOM complex is directly involved in the MOM-targeted integration of PINK1, which is one of the PARK genes and regulating mitochondrial quality control. Upon mitochondrial damage, PINK1 accumulates in the MOM as a protein complex with TOM subunits [46, 47], which was further unveiled crystal clear in a high-resolution atomic model [48]. While the importance of TOM complex in the regulation of mitochondrial quality control has been established, less is known about how the integrity of TOM complex itself is maintained. Given the potential association between TOM and ATP13A2, it is very intriguing that yeast Spf1, ER-resident analogue pump of human ATP13A2, is directly engaged in evulsion of mistargeted Tom20 from ER membrane and resorting to the MOM [45]. Since the current study similarly pointed to interaction between ATP13A2 and Tom20, it is tempting to speculate that ATP13A2 may be involved in regulation or quality control of TOM complex. For testing such hypothesis, specificity of ATP13A2 interaction, especially potential interaction with other TOM subunits needs to be further investigated.

## Conclusions

Localization of overexpressed TOM20, a mitochondrial protein, is influenced by expressing a lysosomal pump, ATP13A2. ATP13A2 expression altered localization of mCherry-TOM20-N to merge with ATP13A2-positive autolysosome. This process, sensitive to MPP^+^, may potentially contribute to maintaining the local proteostasis on the outermembrane of mitochondria.

## Supporting information

S1 Fig Full documentation of the immunostaining data (ATP13A2-Halo). Only representative images are shown in the main Fig 2.
(PDF)

S2 Fig Scatter plots of the images used in the main Fig 1A and 1B. Representative 3 plots for each experimental condition are shown. A, ATP13A2-Halo-OregonG vs LysoTracker. B, ATP13A2-Halo-OregonG vs MitoTracker.
(PDF)

S3 Fig Scatter plots (Mito Grx1-roGFP2 vs mCherry-Tom20-N) of the images used in the main Fig 3B and 3C. Representative 3 plots for each experimental condition are shown. Cells were co-transfected with ATP13A2-Halo (B), resulting in partial segregation of Tom20 signals from Mito Grx1-roGFP2 signals (dashed circles). Without ATP13A2 co-transfection (A), Mito Grx1-roGFP2 and mCherry-Tom20-N signals produced practically perfect overlap.
(PDF)

S4 Fig Scatter plots of the images used in the main Fig 3D and 3E. Representative 3 plots for each experimental condition are shown. A, Mito Grx1-roGFP2 vs ATP13A2-Halo-TMR. B, ATP13A2-Halo-OregonG vs mCherry-Tom20-N. C, ATP13A2(D513A)-Halo-OregonG vs mCherry-Tom20-N.
(PDF)

S5 Fig ATP13A2 overexpression altered the intracellular distribution of GFP-Mff (mitochondrial outermembrane) in HeLa cells. GFP-Mff and ATP13A2-Halo were cotransfected and ATP13A2-Halo was labeled by TMR ligand, followed by live-cell imaging.
(PDF)

S6 Fig Scatter plots of the images used in the main Fig 4A and 4B. Representative 3 plots for each experimental condition are shown. Cells were incubated in the presence of 500 μM MPP+ for 24 hours (upper panel). For comparison, data for normal conditions (the same as the plots shown in B in S4 Fig) are shown (lower panel).
(PDF)

S7 Fig Original images of the membranes shown in the main Fig 6. Nonspecific bands are marked with asterisks.
(PDF)
