# Supplementary material for: ATP13A2 modifies mitochondrial localization of overexpressed TOM20 to autolysosomal pathway
Source: PLoS One. 2022 Nov 29;17(11):e0276823. doi: 10.1371/journal.pone.0276823 (PMC9707766; doi:10.1371/journal.pone.0276823)
Supplement: S1 Fig — Only representative images are shown in the main Fig 2. (PDF) [file pone.0276823.s001.pdf]

Supplementary Data

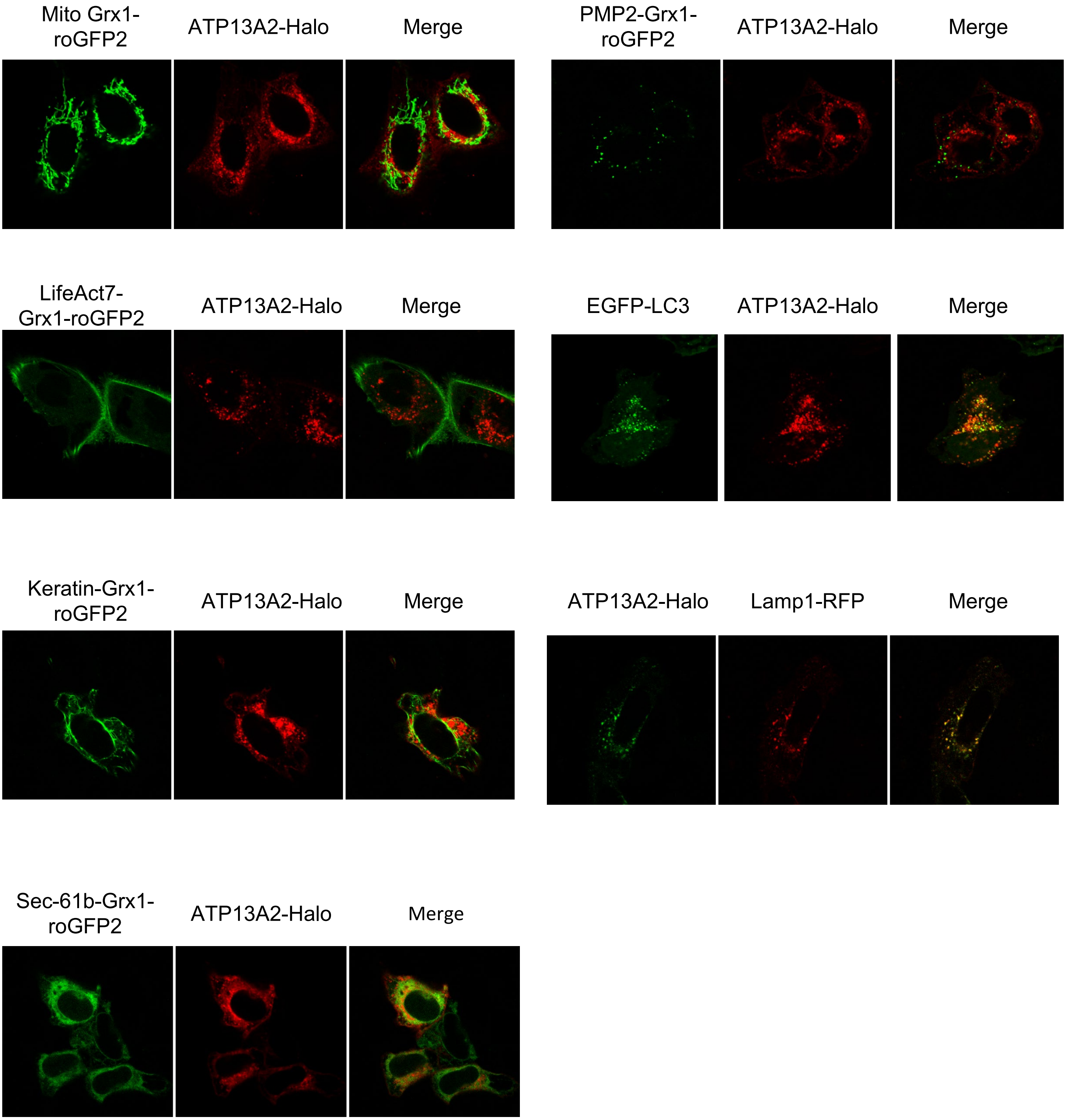

S1 Fig. Full documentation of the immunostaining data (ATP13A2-Halo). Only representative images are shown in the main figure 2.
