# Supplementary material for: ATP13A2 modifies mitochondrial localization of overexpressed TOM20 to autolysosomal pathway
Source: PLoS One. 2022 Nov 29;17(11):e0276823. doi: 10.1371/journal.pone.0276823 (PMC9707766; doi:10.1371/journal.pone.0276823)
Supplement: S2 Fig — Representative 3 plots for each experimental condition are shown. A, ATP13A2-Halo-OregonG vs LysoTracker. B, ATP13A2-Halo-OregonG vs MitoTracker. (PDF) [file pone.0276823.s002.pdf]

A

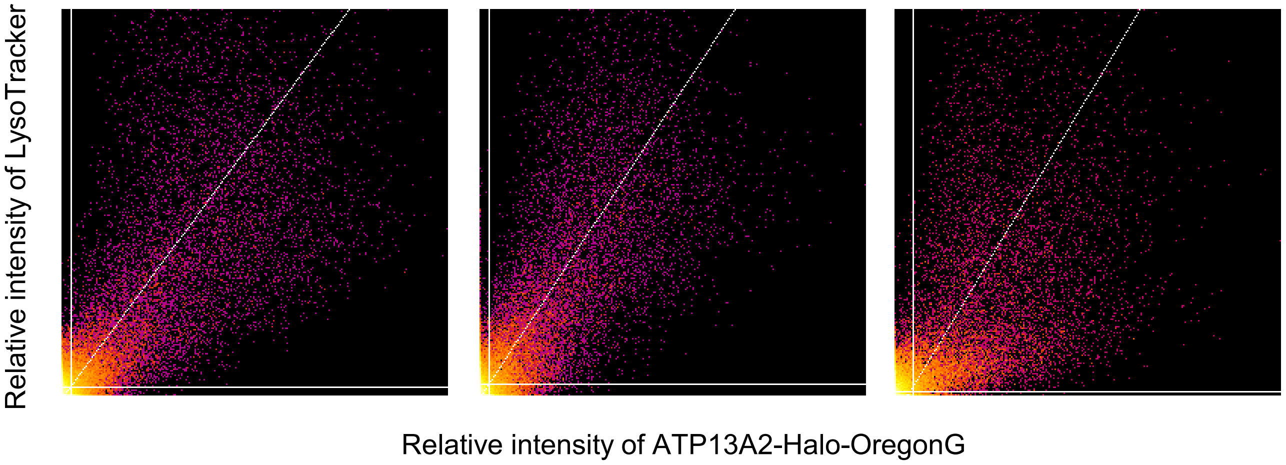

B

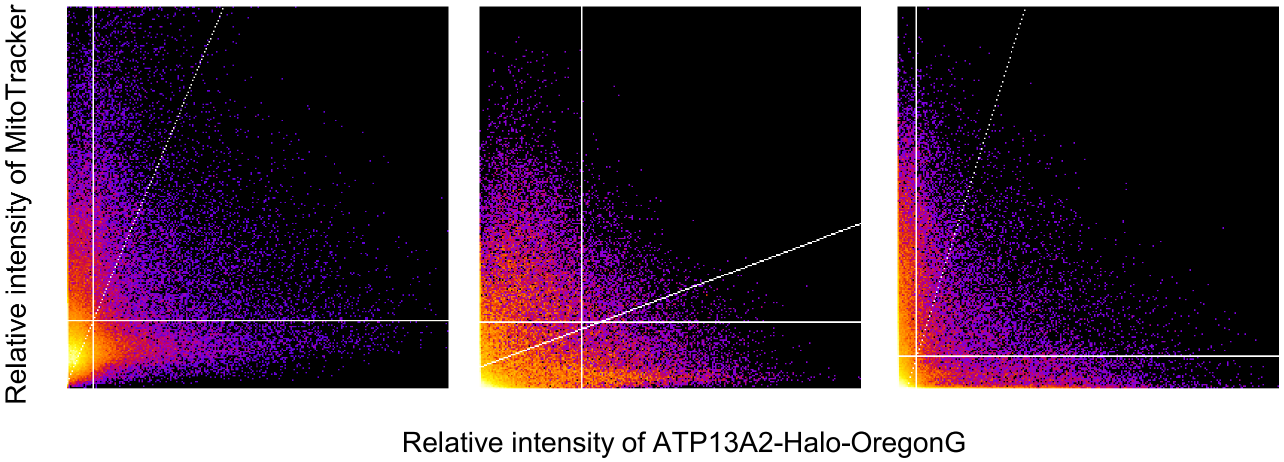

S2 Fig. Scatter plots of the images used in the main figures 2A and B. Representative 3 plots for each experimental condition are shown. A, ATP13A2-Halo-OregonG vs LysoTracker. B, ATP13A2-Halo-OregonG vs MitoTracker.
