# Supplementary material for: ATP13A2 modifies mitochondrial localization of overexpressed TOM20 to autolysosomal pathway
Source: PLoS One. 2022 Nov 29;17(11):e0276823. doi: 10.1371/journal.pone.0276823 (PMC9707766; doi:10.1371/journal.pone.0276823)
Supplement: S3 Fig — Representative 3 plots for each experimental condition are shown. Cells were co-transfected with ATP13A2-Halo (B), resulting in partial segregation of Tom20 signals from Mito Grx1-roGFP2 signals (dashed circles). Without ATP13A2 co-transfection (A), Mito Grx1-roGFP2 and mCherry-Tom20-N signals produced practically perfect overlap. (PDF) [file pone.0276823.s003.pdf]

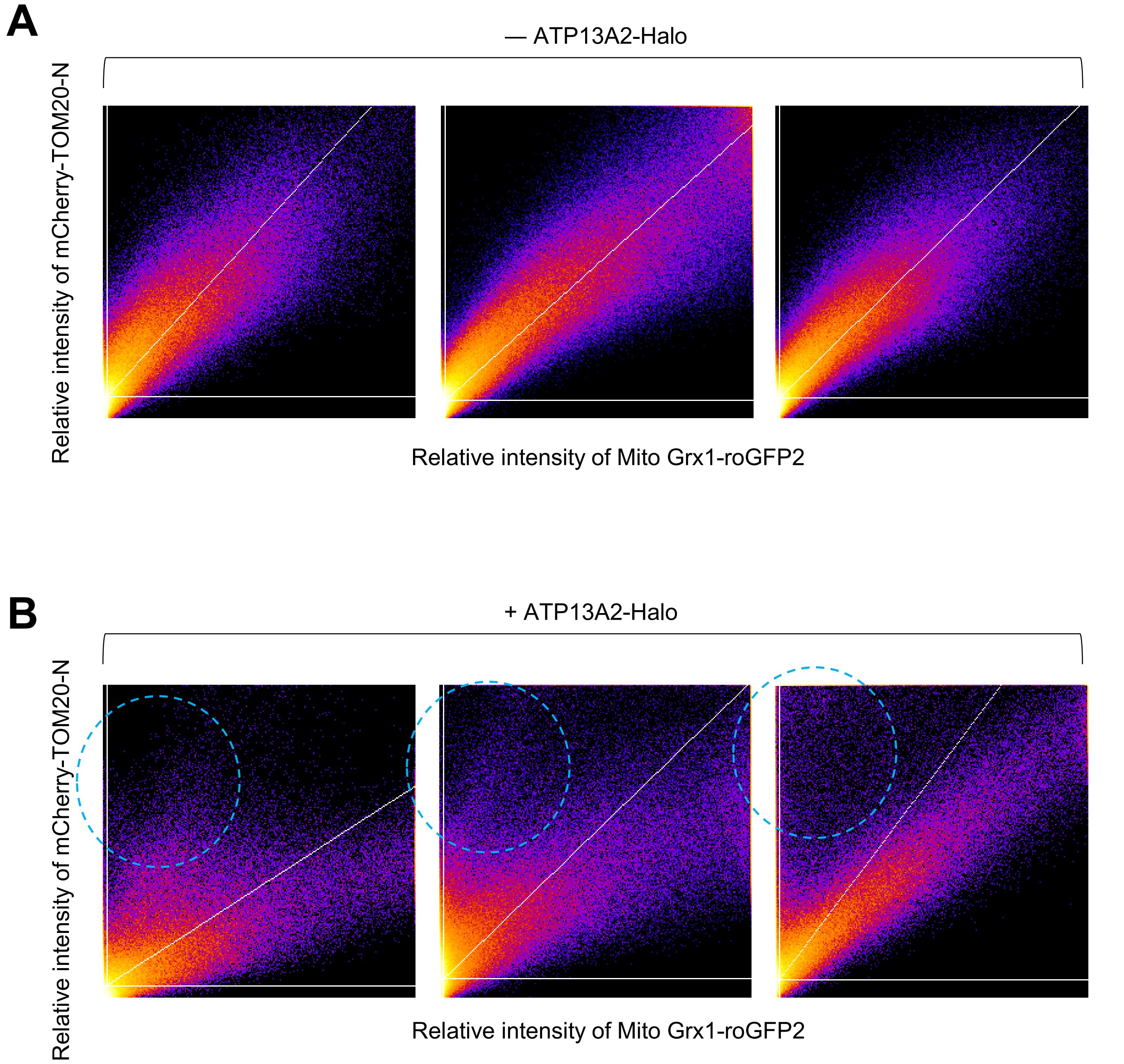

S3 Fig. Scatter plots (Mito Grx1-roGFP2 vs mCherry-Tom20-N) of the images used in the main figure 3B and 3C. Representative 3 plots for each experimental condition are shown. Cells were co-transfected with ATP13A2-Halo (B), resulting in partial segregation of Tom20 signals from Mito Grx1-roGFP2 signals (dashed circles). Without ATP13A2 co-transfection (A), Mito Grx1-roGFP2 and mCherry-Tom20-N signals produced practically perfect overlap.
