# Supplementary material for: ATP13A2 modifies mitochondrial localization of overexpressed TOM20 to autolysosomal pathway
Source: PLoS One. 2022 Nov 29;17(11):e0276823. doi: 10.1371/journal.pone.0276823 (PMC9707766; doi:10.1371/journal.pone.0276823)
Supplement: S4 Fig — Representative 3 plots for each experimental condition are shown. A, Mito Grx1-roGFP2 vs ATP13A2-Halo-TMR. B, ATP13A2-Halo-OregonG vs mCherry-Tom20-N. C, ATP13A2(D513A)-Halo-OregonG vs mCherry-Tom20-N. (PDF) [file pone.0276823.s004.pdf]

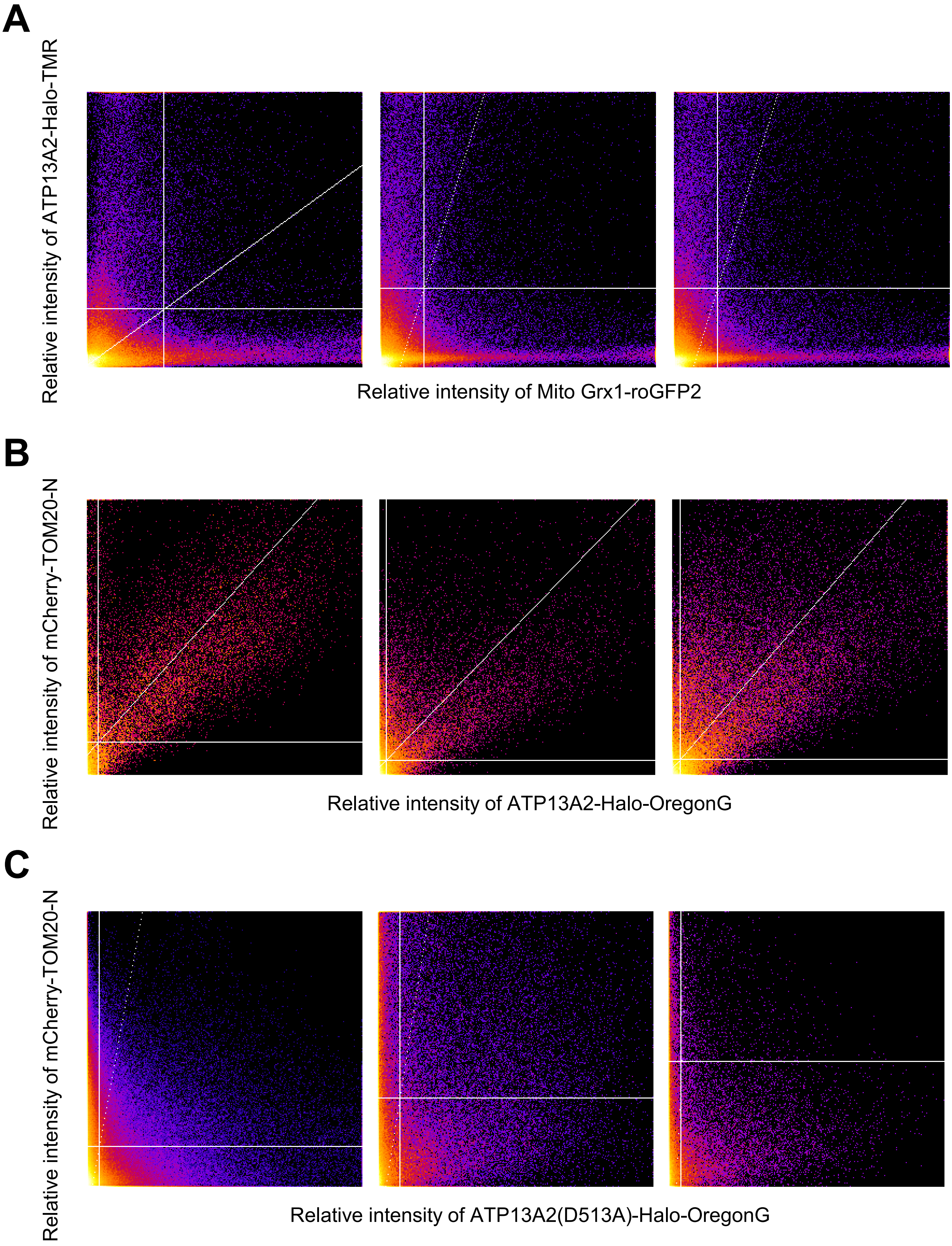

S4 Fig. Scatter plots of the images used in the main figure 3D and 3E. Representative 3 plots for each experimental condition are shown. A, Mito Grx1-roGFP2 vs ATP13A2-Halo-TMR. B, ATP13A2-Halo-OregonG vs mCherry-Tom20-N. C, ATP13A2(D513A)-Halo-OregonG vs mCherry-Tom20-N.
