# Supplementary material for: ATP13A2 modifies mitochondrial localization of overexpressed TOM20 to autolysosomal pathway
Source: PLoS One. 2022 Nov 29;17(11):e0276823. doi: 10.1371/journal.pone.0276823 (PMC9707766; doi:10.1371/journal.pone.0276823)
Supplement: S5 Fig — GFP-Mff and ATP13A2-Halo were cotransfected and ATP13A2-Halo was labeled by TMR ligand, followed by live-cell imaging. (PDF) [file pone.0276823.s005.pdf]

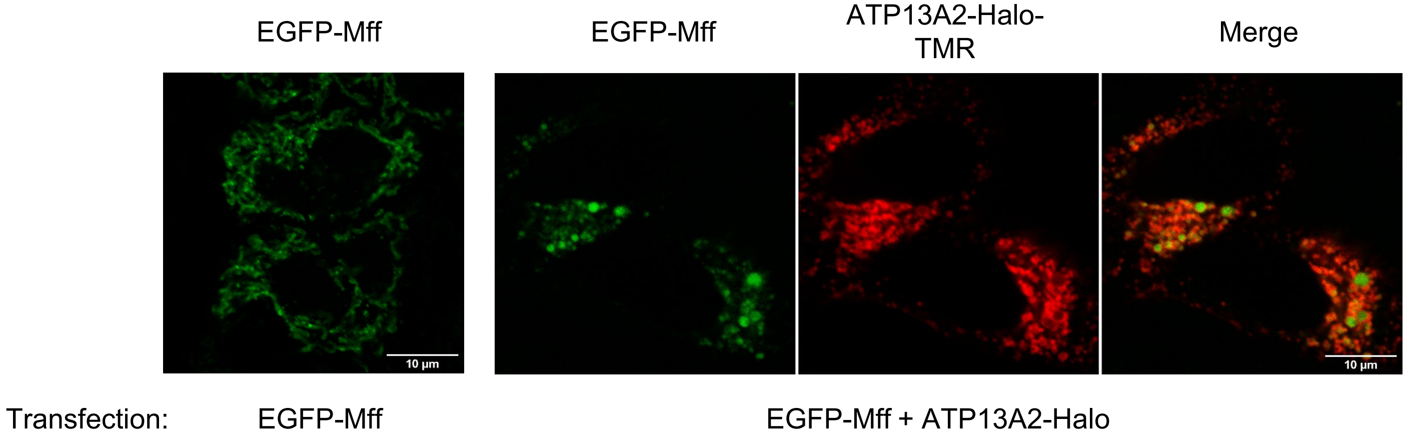

S5 Fig. ATP13A2 overexpression altered the intracellular distribution of GFP-Mff (mitochondrial outermembrane) in HeLa cells. GFP-Mff and ATP13A2-Halo were cotransfected and ATP13A2-Halo was labeled by TMR ligand, followed by live-cell imaging.
