# Supplementary material for: ATP13A2 modifies mitochondrial localization of overexpressed TOM20 to autolysosomal pathway
Source: PLoS One. 2022 Nov 29;17(11):e0276823. doi: 10.1371/journal.pone.0276823 (PMC9707766; doi:10.1371/journal.pone.0276823)
Supplement: S6 Fig — Representative 3 plots for each experimental condition are shown. Cells were incubated in the presence of 500 μM MPP+ for 24 hours (upper panel). For comparison, data for normal conditions (the same as the plots shown in B in S4 Fig) are shown (lower panel). (PDF) [file pone.0276823.s006.pdf]

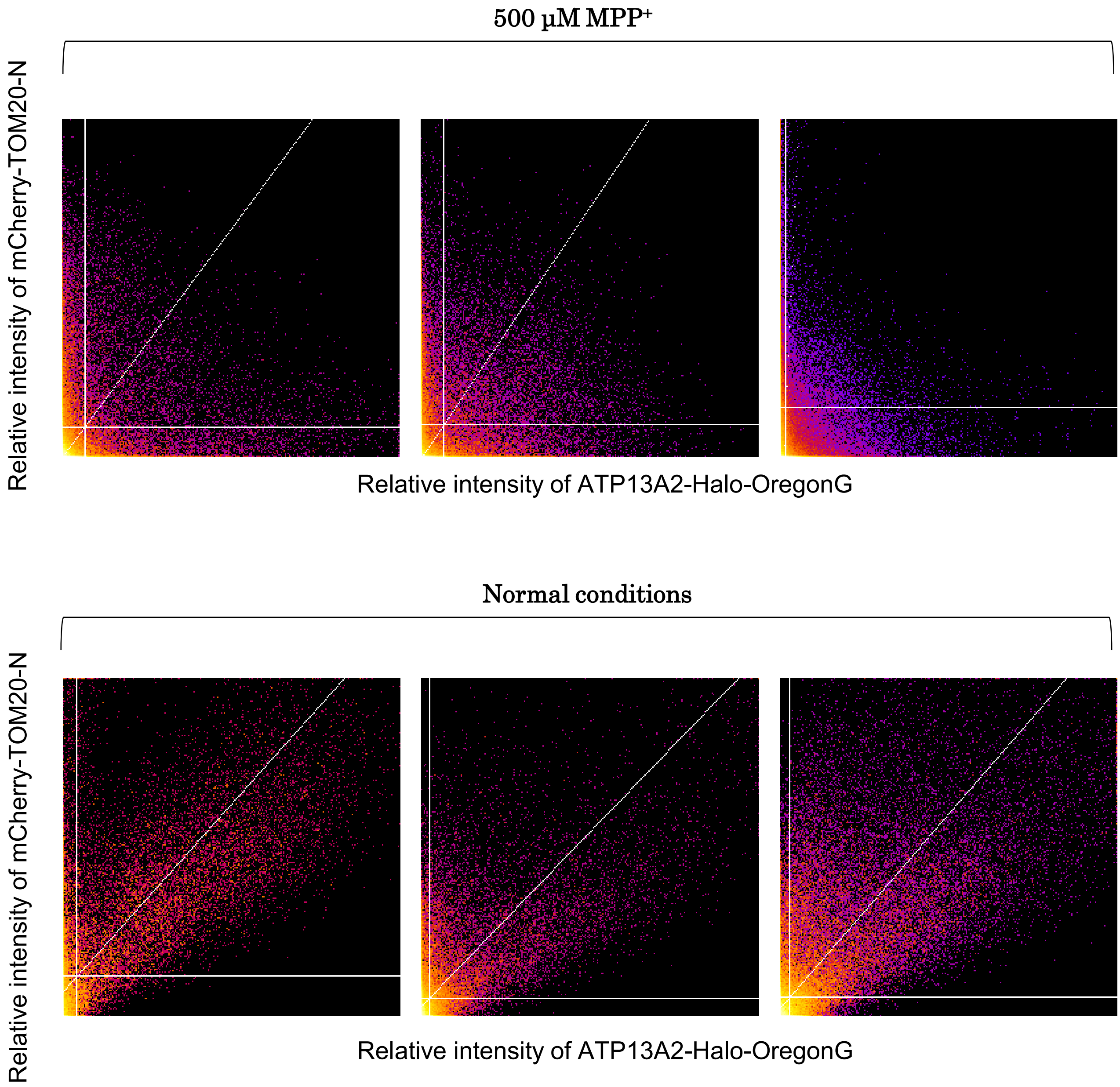

S6 Fig. Scatter plots of the images used in the main figure 4A and B. Representative 3 plots for each experimental condition are shown. Cells were incubated in the presence of 500 µM MPP^+ for 24 hours (upper panel). For comparison, data for normal conditions (the same as the plots shown in supplementary figure 4B) are shown (lower panel)
