# Supplementary material for: ATP13A2 modifies mitochondrial localization of overexpressed TOM20 to autolysosomal pathway
Source: PLoS One. 2022 Nov 29;17(11):e0276823. doi: 10.1371/journal.pone.0276823 (PMC9707766; doi:10.1371/journal.pone.0276823)
Supplement: S7 Fig — Nonspecific bands are marked with asterisks. (PDF) [file pone.0276823.s007.pdf]

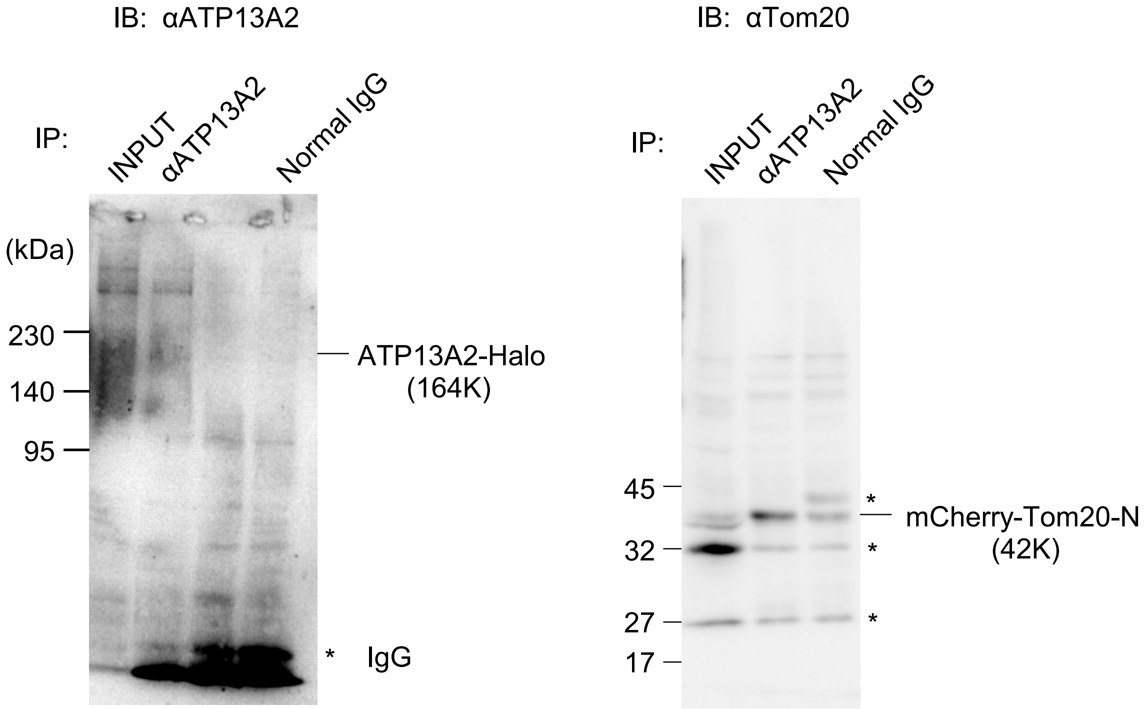

S7 Fig. Original images of the membranes shown in the main figure 6. Nonspecific bands are marked with asterisks.
